# Lysophosphatidic Acid Reduces Ischemic Brain Injury by Attenuating Vascular Permeability Through LPA4 Receptor Signaling

**DOI:** 10.1007/s12975-026-01451-8

**Published:** 2026-06-02

**Authors:** Shintaro Yamada, Kazuhiro Takara, Naoi Hosoe, Anna Shimizu, Yumiko Hayashi, Lamri Lynda, Takayuki Sonoda, Kenichiro Kikuta, Hiroyasu Kidoya

**Affiliations:** 1 Department of Integrative Vascular Biology, Faculty of Medical Sciences, University of Fukui, 23-3 Matsuoka-Shimoaizuki, Eiheiji, Yoshida, Fukui 910-1193 Japan; 2 Department of Neurosurgery, Division of Medicine, Faculty of Medical Sciences, University of Fukui, Fukui, 910-1193 Japan; 3 Tenure-Track Program for Innovative Research, University of Fukui, Fukui, 910-1193 Japan; 4 Otothinolaryngology. Head & Neck Surgery, Faculty of Medicine, University of Fukui, Fukui, 910-1193 Japan

**Keywords:** Lysophosphatidic acid, Blood–brain barrier, Cerebral ischemia, LPA4 receptor, Tight junction, Vascular permeability

## Abstract

Blood–brain barrier (BBB) disruption significantly exacerbates secondary injury following ischemic stroke. Although lysophosphatidic acid (LPA) is known to regulate vascular stability, its specific role in preserving BBB integrity during cerebral ischemia remains unclear. Here, we investigated the neuroprotective effects of LPA using a mouse model of distal middle cerebral artery occlusion (dMCAO). Eight- to ten-week-old female C57BL/6 mice received 10 mg/kg LPA intraperitoneally immediately after ischemia, and outcomes were assessed at 48 h after dMCAO via infarct volumetry, edema quantification, BBB permeability assays (Evans blue and FITC-dextran), and transcriptomic analyses. We demonstrate that LPA treatment significantly reduced infarct volume by approximately 60% and attenuated cerebral edema at 48 h post-ischemia; This protection was accompanied by preserved BBB integrity and maintained endothelial claudin-5 expression in the ischemic territory. Single-cell analysis identified selective and sustained expression of the LPA4 receptor in ischemic vascular endothelial cells. Crucially, the protective effects of LPA on infarct size and BBB permeability were abolished in LPA4 receptor-knockout mice. These findings indicate that LPA preserves BBB integrity and mitigates ischemic brain injury via an endothelial LPA4 receptor-dependent mechanism, identifying the LPA–LPA4 signaling axis as a promising therapeutic target for reducing secondary brain injury in ischemic stroke.

## Introduction

Ischemic stroke remains a leading cause of adult disability and mortality worldwide despite advances in acute care and secondary prevention strategies [1]. Following cerebral ischemia, disruption of the blood–brain barrier (BBB) represents a pivotal pathophysiological event [2] that exacerbates secondary brain injury by increasing vascular permeability, promoting inflammatory cell infiltration, and facilitating cerebral edema [3, 4]. The BBB is a highly specialized interface between the circulating blood and the central nervous system, maintained by brain-endothelial tight junction proteins such as claudin-5, occludin, and zonula occludens-1 (ZO-1) [5–7]. BBB breakdown after ischemia occurs in a biphasic manner—an early disruption within hours and a delayed phase spanning several days—allowing blood-borne molecules and inflammatory cells to enter the brain parenchyma [3, 8–10]. Preserving BBB integrity has, thus, emerged as a promising therapeutic strategy to limit neuronal loss and improve functional outcomes after stroke.

Lysophosphatidic acid (LPA) is a bioactive phospholipid mediator that signals through at least six G protein-coupled receptors (LPA1–LPA6) to regulate diverse cellular functions, including proliferation, migration, and cytoskeletal rearrangements [11–13]. In the nervous system, LPA signaling influences neurogenesis, neuropathic pain, and neuroinflammation [14], and its effects in central nervous system (CNS) injury are highly context-dependent—varying with receptor subtype, cell type, and temporal dynamics [14, 15]. Despite these established roles of LPA in CNS physiology and injury, its specific effects on BBB integrity and stroke outcomes, particularly in the context of cerebral ischemia, remain incompletely understood.

LPA can modulate endothelial barrier function in peripheral tissues [16–19], and emerging evidence indicates that LPA receptor signaling contributes to vascular regulation and endothelial barrier stability. In particular, the LPA4 receptor regulates endothelial permeability and vascular integrity across multiple organ systems [20–23]. Because the BBB is formed by a specialized subset of endothelial cells that share core barrier-regulatory mechanisms with peripheral vasculature, these findings raise the possibility that similar LPA4-dependent pathways may operate in the cerebral endothelium. However, whether LPA4 regulates BBB permeability after stroke has not been well characterized. Furthermore, the specific LPA receptor subtypes mediating these effects in the cerebral vasculature remain unknown. Notably, prior studies examining LPA signaling in cerebral microvascular endothelium and ischemia models have predominantly reported increased permeability and detrimental outcomes, which are often attributed to LPA1 or the autotaxin–LPA axis [24–26]. These findings indicate that LPA signaling in the brain vasculature is context-dependent and frequently disrupts the BBB, thereby making a receptor-specific protective role mediated by LPA4 a particularly novel and contrasting hypothesis. Although *LPA4* mRNA expression has been detected in brain tissue [11, 27–29], direct evidence of *LPA4* expression specifically in brain microvascular endothelial cells under normal or ischemic conditions remains lacking. This gap in knowledge is a key obstacle to understanding the receptor-specific role of LPA signaling in BBB regulation and provides a strong rationale for the present study.

Based on these studies, we hypothesized that analogous LPA4-dependent mechanisms modulate BBB integrity after ischemic stroke. To test this hypothesis, we investigated the effects of exogenous LPA administration on BBB integrity and stroke outcomes in a mouse model of focal cerebral ischemia. Using a permanent distal middle cerebral artery occlusion (dMCAO) model, which produces consistent cortical infarcts with minimal mortality, we assessed infarct volume, behavioral recovery, BBB permeability, and vascular integrity markers following LPA treatment. We further evaluated the role of the LPA4 receptor in mediating these effects using LPA4 knockout mice. Through histological analysis, permeability assays, immunohistochemistry, and RNA-sequencing (RNA-seq), we aimed to determine whether LPA–LPA4 signaling influences BBB stability and ischemic injury. Our findings reveal a previously unrecognized protective function of LPA-LPA4 signaling in preserving BBB integrity following cerebral ischemia, suggesting potential therapeutic applications for modulating this pathway in ischemic stroke.

## Methods

### Data Availability

The data supporting the findings of this study are available from the corresponding author upon reasonable request. RNA-seq datasets generated for this work will be deposited in the Gene Expression Omnibus (GEO) database upon acceptance of the manuscript. In addition, the publicly accessible dataset GSE261494 was utilized to assess the expression of LPA receptors in the mouse cortex.

### Animals, Experimental Design, and Exclusion Criteria

Adult female C57BL/6 mice (8–10 weeks old, 20–25 g, Japan SLC, Inc., Hamamatsu, Shizuoka, Japan) and LPA4 receptor knockout mice on a C57BL/6 background were used [21]. The experimental groups were as follows: (1) Sham + vehicle (wild-type [WT]), (2) dMCAO + vehicle (WT), (3) dMCAO + LPA (WT), (4) dMCAO + vehicle (LPA4 KO), and (5) dMCAO + LPA (LPA4 KO). All outcome measures were assessed in WT mice. In LPA4 KO mice, assessment was limited to Evans blue extravasation and infarct volume measurement using 2,3,5-triphenyltetrazolium chloride (TTC) staining. A total of 138 mice were initially included in this study, of which 14 were excluded because they failed to develop infarction on 2,3,5-triphenyltetrazolium chloride (TTC) staining (*n* = 3), exhibited abnormal behavior attributable to basal ganglia involvement (*n* = 9), or died following Evans blue injection (*n* = 2); leaving 124 mice for final analyses. Animals were maintained under controlled housing conditions (12 h light/dark cycle, 22 ± 2 °C, 55 ± 10% humidity) with *ad libitum* access to food and water. All experimental procedures were approved by the Institutional Animal Care and Use Committee of the University of Fukui (approval number: R07013) and conducted in accordance with the ARRIVE guidelines (Animal Research: Reporting of In Vivo Experiments) for animal experimentation.

### Distal Middle Cerebral Artery Occlusion (dMCAO) Model

Focal cerebral ischemia was induced by permanent occlusion of the distal middle cerebral artery as previously described [30]. Briefly, mice were anesthetized intraperitoneally with a three-drug mixture of medetomidine hydrochloride (0.3 mg/kg), midazolam (4 mg/kg), and butorphanol tartrate (5 mg/kg). Body temperature was maintained at 37 ± 0.5 °C using a heating pad throughout the surgery. After a midline skin incision, a 2 × 2 mm craniotomy was created over the left middle cerebral artery (MCA) territory. The dura was carefully removed, and the distal MCA was exposed and permanently occluded using monopolar electrocoagulation (Low Temperature Cautery Kit, Clearwater, Florida, USA; #18019-00). Sham-operated animals underwent an identical surgical procedure without MCA occlusion. Animals were allowed to recover in a temperature-controlled chamber before returning to their home cages.

### Drug Administration

LPA (18:1, 1-oleoyl-2-hydroxy-sn-glycero-3-phosphate, sodium salt, Avanti Polar Lipids, Alabaster, Alabama, USA; #857130P-25 mg) was dissolved in phosphate-buffered saline (PBS) to obtain a final concentration of 1 mg/mL. The dose of 10 mg/kg was selected based on prior studies demonstrating the vascular-protective effects of LPA on mice [21, 22] and based on preliminary dose-ranging experiments testing 3, 5, and 10 mg/kg doses in our laboratory; among these concentrations, 10 mg/kg yielded the most consistent protective effect across outcome measures. The vehicle control consisted of PBS alone, administered at the same volume to control for any solvent effects. LPA or vehicle was administered intraperitoneally at 10 mg/kg (200 µL per mouse) once immediately after dMCAO, twice within 24 h post-d-MCAO (between days 0 and 1), and then twice again between days 1 and 2. Specifically, the five injections were administered at approximately 0, 12, 24, 36, and 48 h after dMCAO induction. All solutions were freshly prepared before each experiment.

### Measurement of Infarct Volume

Infarct volumes were assessed using TTC staining. Following induction of deep anesthesia, Animals were euthanized by cervical dislocation at designated time points. Brains were rapidly removed and fully immersed in 2% TTC solution at 37 °C for 30 min, followed by fixation in 4% paraformaldehyde. After fixation, brains were sliced into 2-mm-thick coronal sections, and images of the stained sections were acquired using a digital camera (SX70HS; Canon, Tokyo, Japan) for quantitative analysis. Infarcted areas were measured using ImageJ software (NIH, USA; version 1.54 g) by an investigator blinded to treatment groups. Infarct volumes were calculated using the following formula:

$$V\:=\:1/3\;h\;(S1\;+\;\surd(S1\;\times\;S2)\;+\;S2)$$

where h represents the slice width (2 mm), and S1, S2 are the cross-sectional areas of the infarct for consecutive slices [31].

### Brain Water Content

Brain edema was assessed by measuring brain water content using the wet-dry weight method. Following the induction of deep anesthesia, mice were euthanized 48 h after dMCAO, and the brains were quickly removed and dissected into ipsilateral and contralateral hemispheres. The wet weight of each hemisphere was immediately measured. The tissues were then dried in a drying oven at 100 °C for 24 h to obtain the dry weight. Brain water content was calculated based on a previous study using the following formula [32]:

$$Brain\;water\;content\;(\%)\;=\;\lbrack(wet\;weight\;-\;dry\;weight)/wet\;weight\rbrack\;\times\;100\%.$$

### Behavioral Assessment

Functional outcome was evaluated using the cylinder test to quantify forelimb use asymmetry. Mice were placed individually in a transparent cylinder (9 cm in diameter, 15 cm in height) and video recorded for 5 min. The number of wall contacts made with the left forelimb (L), right forelimb (R), or both forelimbs simultaneously (both) was scored by an investigator blinded to the experimental groups. Limb preference was calculated following a previously defined method using the following equation [33]:

$$Limb\;preference\;(\%)\;=\;(L-R)/(L\;+\;R\;+\;both)\;\times\;100\%$$

Positive values indicated left forelimb preference, and the negative values indicated right forelimb preference. Assessments were performed at baseline (before surgery) and on days 1, 3, and 7 after dMCAO.

### BBB Permeability Assessment

BBB integrity was evaluated using two complementary approaches. For Evans blue extravasation (Miles assay), mice received an intravenous injection of Evans blue dye (4%, 4 mL/kg; FUJI FILM Wako, Osaka, Japan; #056–04061) 2 h before sacrifice. After euthanization by cervical dislocation, brains were rapidly removed, and accumulation of dye was quantified spectrofluorometrically (excitation 620 nm, emission 680 nm) and expressed as µg/g tissue [34]. To evaluate macromolecular permeability, FITC-dextran (200 kDa, 10 mg/mL, 0.1 mL; Sigma-Aldrich, St. Louis, Missouri, USA; #FD2000s 100 mg) was administered intravenously 15 min before euthanasia. Subsequently, the animals were euthanized by cervical dislocation, and their brains were harvested immediately. After post-fixation, brains were sectioned and imaged using fluorescence microscopy. FITC-dextran extravasation was quantified by measuring fluorescence intensity outside CD31-positive vessels using ImageJ software [35].

### Immunofluorescence Staining

Brain cryosections (40 μm) were blocked with 5% normal goat serum in PBS containing 0.3% Triton X-100 for 1 h at room temperature, followed by overnight incubation at 4 °C with primary antibodies against CD31 (1:200; Rat monoclonal, BD Phamingen, San Diego, California, USA; #553370), claudin-5 (1:200; Rabbit polyclonal, Thermo Fisher Scientific, Waltham, Massachusetts, USA; #34–1600), or MAP2 (1:500, Chicken polyclonal, abcam, Cambridge, England; #ab5393). After washing, sections were incubated with appropriate fluorescent secondary antibodies for 2 h at room temperature. Images were acquired using a Leica Stellaris 5 (Leica Microsystems, Wetzlar, Germany) confocal microscope and analyzed with ImageJ software. For quantification of vessel density, the percentage of CD31 or claudin-5 positive area relative to the total area of the region of interest (located between + 1.0 and − 1.0 mm relative to the bregma) was calculated using the Angio tool [36]. A minimum of three animals per group were included in quantitative analyses. For each animal, four non-overlapping fields of view were acquired from three consecutive brain sections.

### Flow Cytometry

Brain microvascular endothelial cells (BMECs) were isolated from ischemic brain tissue collected 48 h after dMCAO. The isolation of BMECs, cell-surface antigen staining, and flow cytometry were performed following previously described methods. Briefly, the dissected brain tissue was enzymatically digested using collagenase/dispase (1 mg/mL) and DNase I (100 U/mL) at 37˚C for 30 min, followed by mechanical dissociation and density gradient centrifugation (Percoll, 37%/70%) to enrich for microvascular fragments. The resulting cell suspension was then stained with a viability dye and antibodies against CD31 and CD45. BMECs were identified and sorted as CD31 + CD45- live cells using a SONY SH800 Cell Sorter system (SONY, Tokyo, Japan) [37]. Live cells were identified using flow cytometry after staining with Zombie NIR Fixable Viability Kit (BioLegend, San Diego, California, USA; #423105) and labeled with antibodies against CD31 (Rat monoclonal, BioLegend; #102409) and CD45 (Rat monoclonal, BioLegend; #103108). Cells were sorted using a SONY SH800 cell sorter (SONY, Tokyo, Japan) and immediately processed for RNA extraction.

### RNA Extraction

RNA was extracted from brain tissue and BMECs by lysing samples in Buffer RLT, followed by thorough homogenization using a QIAshredder Spin Column (QIAGEN, Hilden, Germany), centrifuged at maximum speed (20,380 × g) for 2 min. Total RNA was then purified using the RNeasy Micro Kit and QIAcube system (QIAGEN) following the manufacturer’s instructions. RNA concentration and RNA integrity number (RIN) were assessed using an Agilent 2100 Bioanalyzer (Agilent Technologies, Santa Clara, CA, USA). Only samples with a RIN > 7.0 were used for subsequent RNA -sequencing.

### RNA-seq and Bioinformatics Analysis

RNA-seq data were analyzed using iDEP (ver2.5.2) (http://bioinformatics.sdstate.edu/idep/). Differentially expressed genes (DEGs) were identified using DESeq with a maximum false discovery rate-adjusted *p* < 0.05, and a 1.5-fold change (FC) between groups [38–40]. The RNA-seq data have been deposited in the GEO database (accession code: PRJDB39906).

### Gene Enrichment Analysis

To characterize the functional pathways associated with transcripts modulated by ischemic stroke and LPA treatment, two gene sets were defined: (i) genes upregulated after dMCAO but downregulated following LPA administration, and (ii) genes downregulated after dMCAO but upregulated with LPA treatment. Gene set enrichment analysis was performed using a comprehensive GMT-formatted collection of curated gene sets derived from publicly available databases, including Reactome, Kyoto Encyclopedia of Genes and Genomes (KEGG, *Homo sapiens*), WikiPathways, Pathway Interaction Database, Integrative Network of Organ System (INOH), BioCarta, and TarBase (miRNA-target gene sets) [41–46].

### Re-analysis of Public Single-cell RNA-seq Data

Single-cell RNA-seq datasets of the mouse brain were downloaded from the GEO database (accession ID: GSE261494) and processed using Seurat (v5.0.0) in R (v4.3.2). Datasets were integrated using the Harmony R package (v1.2.0) to correct for batch effects while preserving biological variability. Uniform Manifold Approximation and Projection (UMAP) was applied for dimensionality reduction and visualization. Cell types were annotated based on canonical marker genes and guided by the classifications in the original publication. Major cell populations included endothelial cells (EC), neurons, oligodendrocytes (Oligo), microglia (MG), astrocytes (Astro), lymphocytes, epithelial cells, vascular leptomeningeal cells, and venous endothelial cells [47].

### Statistical Analysis

All statistical analyses were pre-specified before unblinding. Data are presented as mean ± standard error of the mean (SEM) with individual data points shown. Statistical analyses were performed using GraphPad Prism software (version 9.5, GraphPad Software Inc., San Diego, CA). Normality was assessed using the Shapiro–Wilk test. Differences between the two groups were analyzed using an unpaired two-tailed Student’s t-test. Multiple comparisons were performed using one-way ANOVA followed by Tukey’s post-hoc test or two-way ANOVA followed by Bonferroni’s post-hoc test. Statistical significance was defined as *p* < 0.05.

## Results

### LPA Treatment Reduces Infarct Volume and Improves Behavioral Outcomes Following dMCAO

**Fig. 1:**
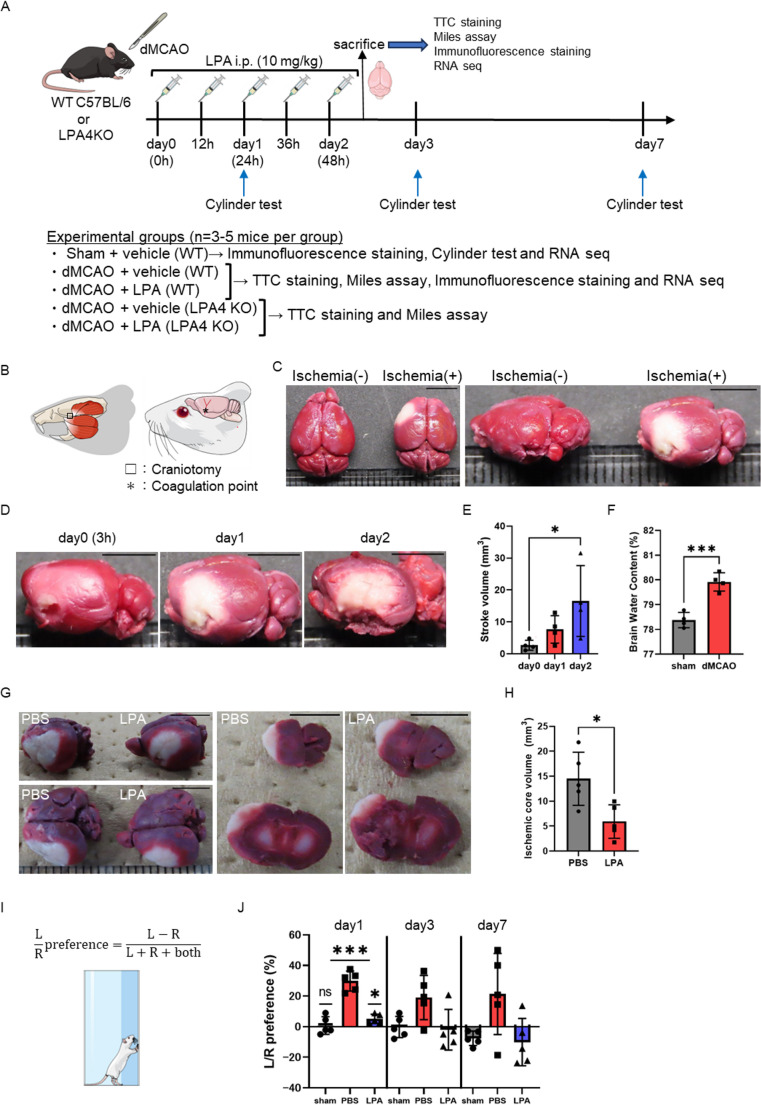
LPA treatment reduces infarct volume in a permanent dMCAO stroke model **(A)** Schematic diagram of the time course of LPA administration and analysis in a model of permanent dMCAO using C57BL/6 and LPA4KO mice. (**B–E**) Characterization of the dMCAO model. **(B)** Schematic of the dMCAO model showing craniotomy site (white box) and coagulation point (asterisk). **(C)** Representative images of ischemic (+) and non-ischemic (-) hemispheres with white areas indicating ischemic core and red areas representing normal tissue. **(D)** Progressive development of the ischemic region at 3 h (day 0), day 1, and day 2 post-dMCAO. **(E)** Quantification of stroke volume (mm³) over time following dMCAO. **(F)** Brain water content (%) comparison between sham-operated and dMCAO groups. **(G)** Representative images comparing ischemic regions in PBS-treated versus LPA-treated brains from multiple viewing angles. **(H)** Quantitative comparison of ischemic core volume (mm³) between PBS and LPA-treated groups. **(I)** Cylinder test schematic for assessing forelimb motor function. L represents left forelimb contacts, R represents right forelimb contacts, and “both” indicates simultaneous use of both forelimbs. **(J)** Cylinder test results showing limb preference percentages on days 1, 3, and 7 post-stroke in sham, PBS-treated, and LPA-treated mice. Statistical analysis: Two-tailed unpaired t-test (F and H), ANOVA, and post-hoc multiple comparison tests (E and J). Data represent mean ± SEM with individual data points shown. *n* = 4 per group for TTC analysis and brain water content (E and F), *n* = 5 per group for TTC analysis (G), *n* = 5 per group for behavioral analysis (I). *p < 0.05 vs. day 0 (E), PBS (H), ***p < 0.01 vs. sham (F), PBS (J). Scale bar = 5 mm (C, D, and G)

To evaluate the neuroprotective potential of LPA in ischemic stroke, we used a dMCAO model in mice. Following dMCAO induction, LPA was administered every 12 h for a total of five doses. The overall experimental timeline, including the time points for various analyses, is detailed in Fig. 1A. As illustrated in Fig. 1B, permanent occlusion of the distal MCA was achieved via craniotomy and electrocoagulation. This procedure consistently produced cortical infarcts within the MCA territory, confirmed by TTC staining. Brain sections demonstrated a clear distinction between the ischemic core (white, TTC-negative) and surrounding viable tissue (red, TTC-positive) at 3 h (day 0), 24 h (day 1), and 48 h (day 2) post-occlusion (Fig. 1C–E). The dMCAO model also resulted in significant cerebral edema, evidenced by increased water content in the ipsilateral hemisphere compared with sham-operated controls (Fig. 1F). Next, we assessed the therapeutic effects of LPA administration. At 48 h post-ischemia, TTC staining demonstrated that LPA-treated mice exhibited significantly reduced infarct volumes compared with PBS-treated control mice (Fig. 1G). Quantitative analysis indicated an approximately 60% reduction in infarct size in the LPA group, supporting a robust neuroprotective effect (Fig. 1H).

To determine whether this anatomical protection translated into functional improvement, we evaluated forelimb asymmetry using the cylinder test. Vehicle-treated dMCAO mice showed pronounced left forelimb preference (ipsilateral to the lesion) at all time points, reflecting impaired right forelimb function. In contrast, LPA-treated mice displayed trends toward improved symmetry at days 1, 3, and 7 post-ischemia (Fig. 1I, J). The cylinder test showed that LPA treatment did not produce significant changes in forelimb preference within the assessed period. Nevertheless, the observed trends toward improved forelimb symmetry suggest possible functional benefits that may require longer follow-up or complementary behavioral assays to detect them more clearly.

### LPA Treatment Alters Gene Expression Profiles in Ischemic Brain Tissue

**Fig. 2:**
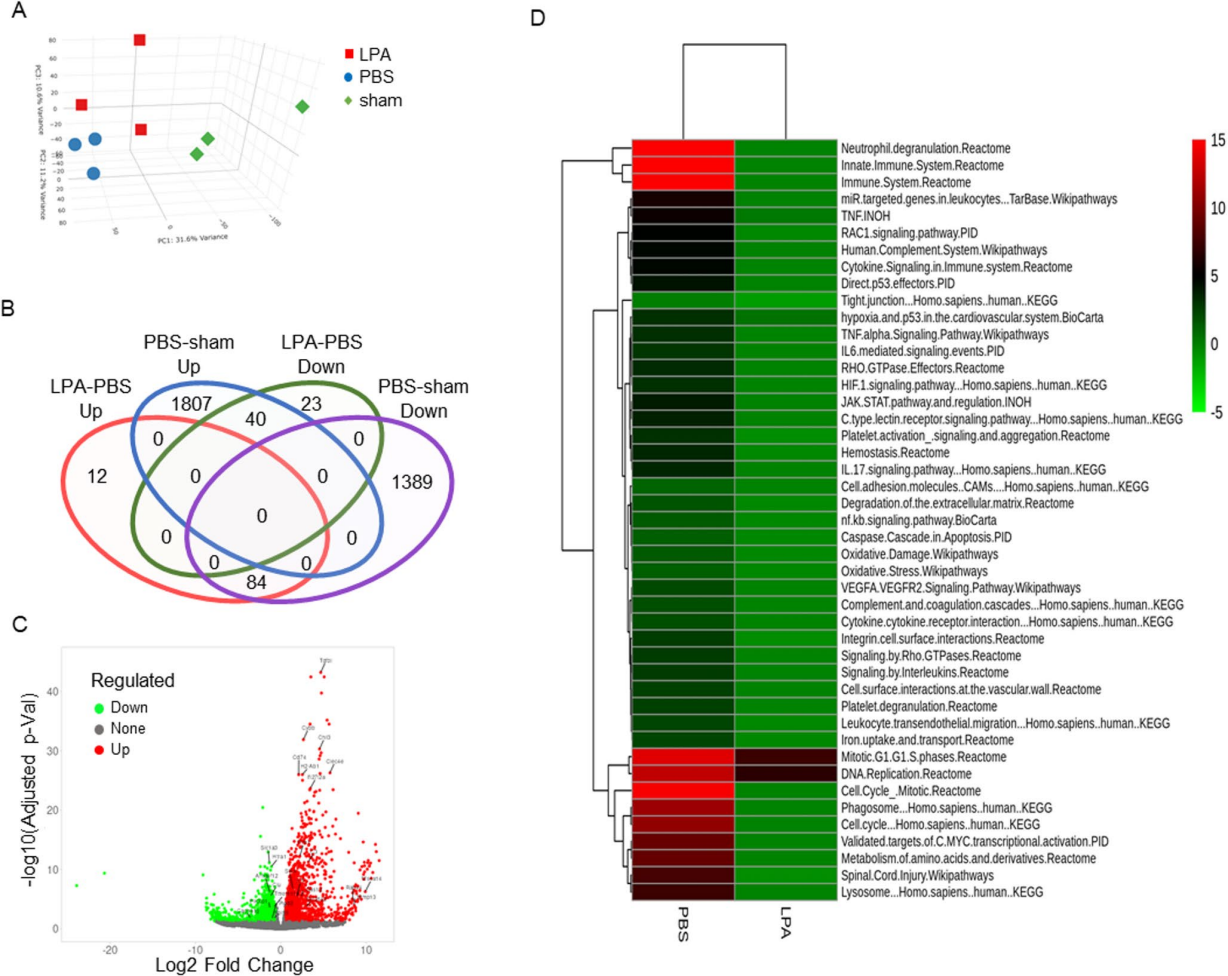
LPA treatment alters gene expression profiles in ischemic brain tissue **A**) Principal component analysis (PCA) of bulk RNA-sequencing (RNA-seq) data from ischemic cortex at 48 h post-dMCAO, showing distinct gene expression patterns among sham, PBS-treated dMCAO, and LPA-treated dMCAO. **B**) Venn diagram analysis of differentially expressed genes (DEGs) in ischemic cortex, comparing PBS-treated dMCAO vs. sham and LPA-treated dMCAO vs. PBS-treated dMCAO. (FDR < 0.05, |fold change| > 1.5). **C**) Volcano plot showing upregulated (red) and downregulated (green) DEGs upon LPA-treated dMCAO (left) and PBS-treated dMCAO (right). Labeled genes represent transcripts upregulated by PBS-treated dMCAO but downregulated by LPA-treated dMCAO, or downregulated by PBS-treated dMCAO but restored/upregulated by LPA-treated dMCAO. **D**) Heatmap showing the activation of significantly enriched pathways, comparing PBS-treated dMCAO with LPA-treated dMCAO. Upregulated pathway (red) includes blood vessel morphogenesis, angiogenesis, cell migration, and extracellular matrix organization. Downregulated pathways (green) include inflammatory response, leukocyte migration, and cytokine-mediated signaling. *n* = 3 per group for RNA-seq and bioinformatics analysis

To investigate the molecular mechanisms underlying LPA-mediated neuroprotection, we performed bulk RNA-seq on ischemic cortical tissue collected from sham-operated, PBS-treated dMCAO, and LPA-treated dMCAO mice at 48 h post-ischemia. Principal component analysis (PCA) revealed distinct transcriptional profiles across the three groups **(**Fig. 2A), with Sham and ischemic samples separated along PC1. In contrast, PBS- and LPA-treated dMCAO samples segregated along PC2, indicating that LPA elicits marked gene expression changes within the injured cortex. Differential expression analysis identified 1,847 DEGs upregulated in PBS-treated dMCAO tissue relative to sham, with 40 of these genes overlapping with those downregulated in the LPA-treated dMCAO group compared with the PBS control group (Fig. 2B). Conversely, among genes downregulated in PBS-treated dMCAO group versus sham group (1,473 genes), 84 overlapped with those upregulated following LPA treatment. These reciprocal patterns highlight subsets of ischemia-responsive genes that LPA modulates. Volcano plot visualization further illustrated the magnitude and significance of LPA-induced transcriptional changes (Fig. 2C), revealing a substantial number of genes exhibiting |log_2_(FC)| > 2 and adjusted p-values < 0.001. Gene ontology (GO) enrichment analysis of LPA-responsive DEGs demonstrated significant overrepresentation of biological processes involved in vascular remodeling, inflammatory signaling, and cell adhesion (Fig. 2D). Specifically, upregulated pathways included blood vessel morphogenesis, angiogenesis, cell migration, and extracellular matrix organization, whereas downregulated pathways were enriched for inflammatory responses, leukocyte trafficking, and cytokine-mediated signaling. Together, these findings indicate that LPA modulates multiple transcriptional programs associated with BBB stabilization and post-ischemic tissue repair.

### LPA Treatment Preserves BBB Integrity After Cerebral Ischemia

**Fig. 3:**
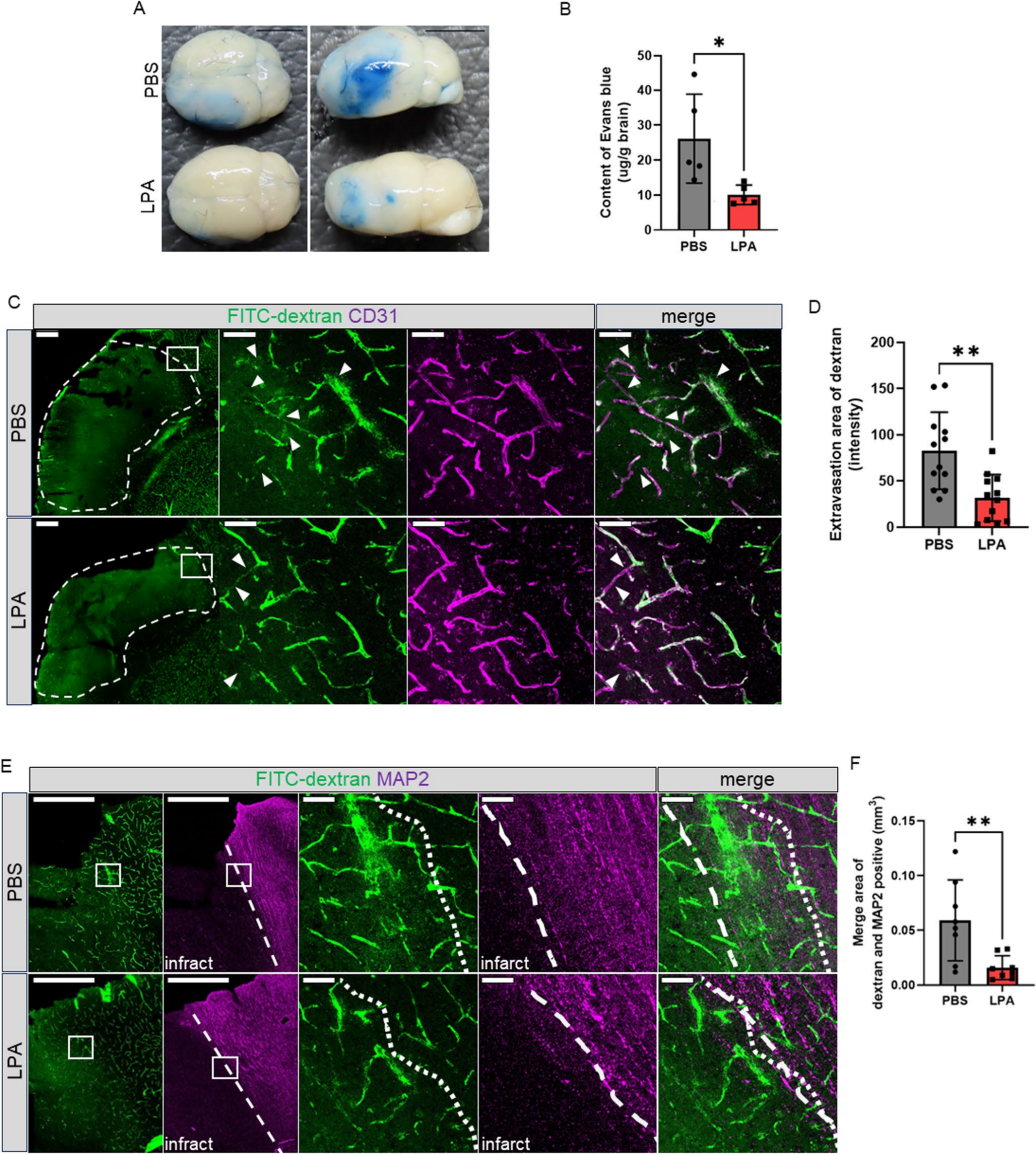
LPA treatment preserves BBB integrity and reduces vascular permeability following cerebral ischemia **A**) Miles assay of blood–brain barrier permeability showing Evans blue extravasation in PBS control versus LPA-treated (10 mg/kg) brains at 48 h post-dMCAO. **B**) Quantification of Evans blue content (µg/g brain tissue) in PBS and LPA-treated brains. **C**) Immunofluorescence images showing FITC-dextran (200 kDa, green) extravasation and CD31-positive blood vessels (magenta) in the ischemic territory. White arrowheads in higher magnification images of the ischemia territory indicate fluorescent signal accumulating outside CD31-positive vessels. **D**) Quantification of FITC-dextran extravasation area (intensity) in the ischemic core of PBS and LPA-treated brains. **E**) Dual immunofluorescence staining for FITC-dextran (green) and MAP2 neuronal marker (magenta) in the peri-infarct (penumbra) region. Higher magnification images comparing FITC-dextran extravasation relative to MAP2-positive neuronal regions in PBS and LPA-treated brains. The area between dotted lines represents the penumbral region where neurons remain viable but are at risk. **F**) Quantification of the overlap area (mm²) between dextran extravasation and MAP2-positive regions in vehicle and LPA groups. Statistical analysis: Two-tailed unpaired t-test (B, D, and F). Data represent mean ± SEM with individual data points shown. *n* = 5 per group for Miles assay (B), *n* = 12 per group for extravasation area of dextran (D), *n* = 8 per group for merge area of dextran and MAP2 positive (F). *p < 0.05 vs. PBS, **p < 0.01 vs. PBS. Scale bar = 5 mm (A), 500 μm (low-magnification of B and C), and 50 μm (high-magnification of B and C)

Given the contribution of BBB disruption to secondary injury following stroke and the transcriptional changes observed in vascular-related pathways, we next examined whether LPA treatment alters BBB permeability after dMCAO. At 48 h post-ischemia, the Miles assay demonstrated significantly reduced Evans Blue dye leakage in the ipsilateral hemisphere of LPA-treated mice compared with that in PBS-treated control mice (Fig. 3A, B), indicating attenuation of BBB disruption.

To more precisely assess BBB permeability, FITC-dextran extravasation was examined in brain sections following dMCAO. Vehicle-treated dMCAO mice exhibited prominent FITC-dextran leakage throughout the ischemic territory. Fluorescent signals were detected in the parenchyma outside CD31-positive vessels (Fig. 3C; white arrowheads). LPA treatment markedly reduced FITC-dextran leakage, and quantitative analysis confirmed significantly reduced extravasation in the ischemic core compared with control treatments (Fig. 3D).

To further investigate the relationship between microvascular protection and neuronal preservation in the peri-infarct (penumbra) region, we performed dual immunofluorescence staining for FITC-dextran and MAP2 at 48 h post-ischemia. In PBS-treated mice, extensive FITC-dextran leakage coincided with areas of diminished MAP2 immunoreactivity in the penumbra (Fig. 3E), consistent with compromised neurovascular unit integrity. In contrast, LPA-treated mice exhibited substantially reduced FITC-dextran extravasation together with better preserved MAP2 staining. Quantification of the area in which dextran signal overlapped with MAP2-positive tissue showed a significant reduction in LPA-treated mice (Fig. 3F), indicating that LPA preserves vascular barrier function in regions containing viable neuronal tissue. Collectively, these findings demonstrate that LPA treatment reduces BBB hyperpermeability in both the ischemic core and the penumbra, supporting a broad protective effect on the neurovascular unit following cerebral ischemia.

### LPA Treatment Maintains Tight Junction Protein Expression in Peri-infarct Regions

**Fig. 4:**
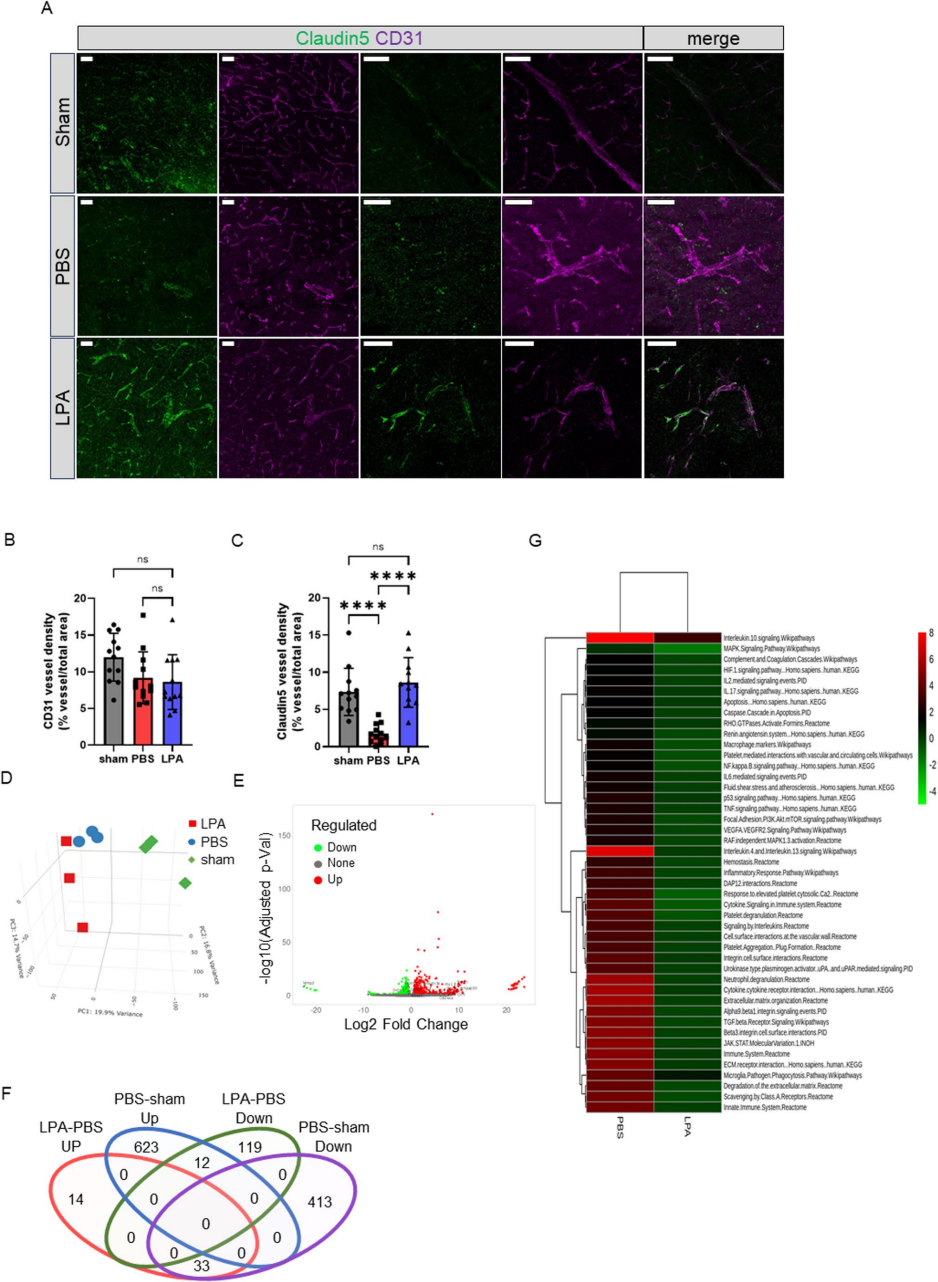
LPA treatment maintains tight junction protein expression in the infarct cerebral vasculature and modulates gene expression in the infarct core. **A**) High-magnification confocal microscopic images displaying claudin-5 expression (green) and CD31 endothelial marker (magenta). The merged images show their spatial relationship in sham, PBS, and LPA-treated brain sections. **B**) Quantification of CD31-positive vessel density (% vessel area/total area) in the sham, PBS, and LPA groups. **C**) Quantification of claudin-5-positive vessel density (% vessel area/total area) in the sham, PBS, and LPA groups. **D**) Principal component analysis (PCA) of single-cell RNA sequencing data from vascular endothelial cells (VEC) isolated from ischemic cortex at 48 h post-dMCAO, showing distinct gene expression patterns among sham, PBS-treated dMCAO, and LPA-treated dMCAO mice. **E**) Volcano plot showing upregulated (red) and downregulated (green) DEGs upon LPA-treated VEC (left) and PBS-treated VEC (right). Labeled genes represent transcripts upregulated by PBS-treated VEC but downregulated by LPA-treated VEC, or downregulated by PBS-treated VEC but restored/upregulated by LPA-treated VEC. **F**) Venn diagram analysis of differentially expressed genes (DEGs) in VEC, comparing PBS-treated VEC vs. sham VEC and LPA-treated VEC vs. PBS-treated VEC (FDR < 0.05, |fold change| > 1.5). **G**) Heatmap showing the activation of significantly enriched pathways, comparing PBS-treated VEC with LPA-treated VEC. Revealing pathways related to cell adhesion, extracellular matrix organization, and vascular development, consistent with bulk RNA-seq findings. Statistical analysis: analysis of variance (ANOVA), and post-hoc multiple comparison tests (**B** and **C**). Data represent means ± SEM with individual data points shown. *n* = 12 per group for vascular density (B and C), *n* = 3 per group for RNA sequencing and bioinformatics analysis. ****p < 0.0001 vs. sham and PBS groups, ns: not significant (B). Scale bar = 50 μm (A)

Because BBB integrity relies heavily on endothelial tight junctions, we examined whether the protective effects of LPA involve preservation of tight junction protein expression in the peri-infarct region. Immunofluorescence staining for the endothelial marker CD31 and the tight junction protein claudin-5 was performed at 48 h post-ischemia. Compared with those in sham controls, CD31-positive vessels in PBS-treated dMCAO mice exhibited markedly reduced claudin-5 immunoreactivity, consistent with tight junction disruption following ischemic injury. In contrast, LPA-treated mice displayed robust claudin-5 staining along CD31-positive vessels, indicating preservation of tight junction integrity, with no significant differences when compared with sham controls (Fig. 4A).

Quantitative analysis showed no significant difference in CD31-positive vessel density between the three groups (Fig. 4B), suggesting that LPA does not alter overall vascular density within the peri-infarct region. However, claudin-5–positive vessel density was significantly higher in LPA-treated mice compared with PBS-treated controls (Fig. 4C; *P* < 0.05), confirming that LPA attenuates the ischemia-induced loss of claudin-5 expression. Together, these findings indicate that LPA preserves BBB integrity by maintaining tight junction protein expression rather than by inducing angiogenesis or increasing vessel density.

### LPA Treatment Modulates Gene Expression in the Infarct Core

To further investigate LPA-mediated molecular responses within the infarct core, we performed RNA-seq on tissue isolated specifically from this region in LPA-treated and PBS-treated dMCAO mice. PCA analysis demonstrated clear separation between treatment groups (Fig. 4D), indicating distinct transcriptional profiles associated with LPA exposure. Consistent with this, volcano plot analysis revealed numerous significantly regulated genes (Fig. 4E), further supporting robust LPA-dependent transcriptional modulation. Venn diagram analysis identified 635 genes uniquely upregulated and 446 genes uniquely downregulated in PBS-treated samples compared with sham tissue. Among the 635 upregulated genes in the PBS group, 12 overlapped with genes that were downregulated in the LPA-treated dMCAO group compared with the PBS control group (Fig. 4F). GO enrichment analysis of DEGs revealed significant enrichment of pathways associated with cell adhesion, extracellular matrix organization, and vascular development (Fig. 4G), consistent with the bulk RNA-seq findings from the whole cortex and supporting the interpretation that LPA influences vascular function through transcriptional mechanisms rather than structural changes.

### LPA4 Receptor Mediates the BBB-Protective Effects of LPA

**Fig. 5:**
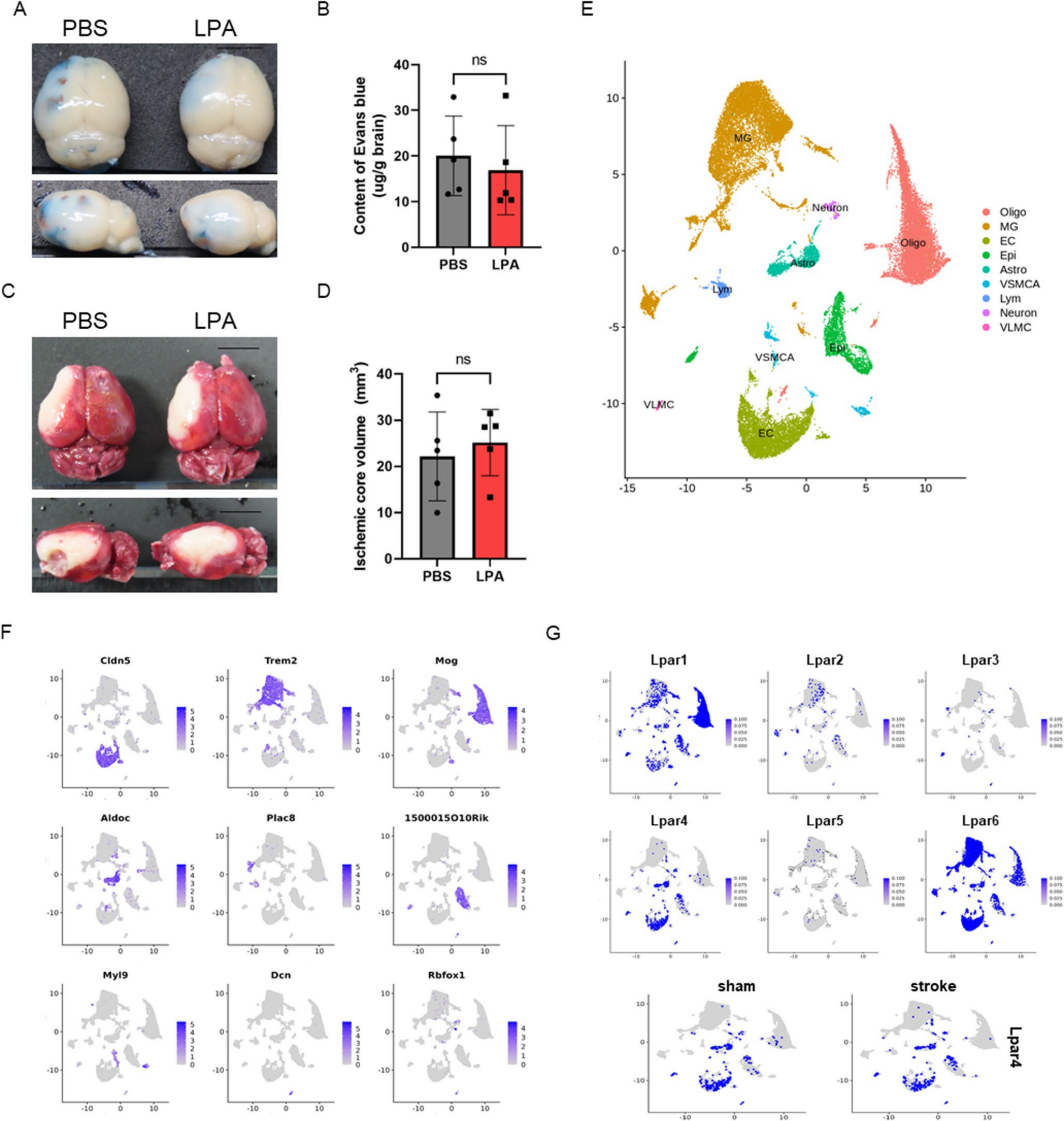
LPA4 receptor mediates BBB-protective effects and is expressed in vascular endothelium **A**) Representative images of Evans Blue extravasation in LPA4 receptor knockout (LPA4 KO) mice treated with PBS or LPA. **B**) Quantification of Evans Blue content in LPA4 KO mice. **C**) Representative TTC-stained brain sections from LPA4 KO mice treated with PBS or LPA. **D**) Quantification of infarct volume in LPA4 KO mice. Scale bar = 5 mm. **E**) UMAP visualization of single-cell RNA sequencing data from ischemic brain tissue at 48 h post-dMCAO, showing graph-based clusters annotated to major cell types using canonical markers. Clusters were annotated to major cell types according to the original publication: endothelial cells (EC), oligodendrocytes (Oligo), microglia (MG), astrocytes (Astro), lymphocytes (Lym), epithelial cells (Epi), vascular leptomeningeal cells (VLMC), venous vascular endothelial cells (VECV). **F**) Feature plots of representative marker genes used for annotation: Cldn5 (EC), Trem2 (MG), Mog (Oligo), Aldoc (Astro), Plac8 (Lym), 1500015O10Rik (Epi), Myl9 (VSMC/arterial), Dcn (VLMC), Rbfox1 (neuron). **G**) LPA4 receptor expression in vascular endothelium is maintained even after ischemic injury, indicating sustained receptor availability for therapeutic targeting. Statistical analysis: Two-tailed unpaired t-test (B and D). Data represent mean ± SEM with individual data points shown. *n* = 5 per group for Miles assay and TTC analysis. ns: not significant (B and D). Scale bar = 5 mm (A and C)

To identify the LPA receptor subtype responsible for the BBB-protective effects of LPA, we evaluated LPA treatment in LPA4 receptor knockout (LPA4 KO) mice subjected to dMCAO. The protective effect of LPA on BBB permeability was abolished in LPA4 KO mice, as evidenced by comparable Evans blue extravasation between LPA-treated and vehicle-treated LPA4 KO mice (Fig. 5A, B). In contrast, wild-type mice showed a reduction in Evans Blue leakage following LPA administration. Evans Blue leakage in LPA-treated LPA4 KO mice remained comparable to vehicle-treated KO controls, indicating loss of LPA-mediated BBB protection. Similarly, LPA treatment failed to reduce infarct volume in LPA4 KO mice (Fig. 5C, D), whereas wild-type mice displayed the expected decrease in infarct size with LPA administration. These findings demonstrate that LPA4 signaling is required for both BBB protection and infarct volume reduction induced by LPA following cerebral ischemia.

### LPA4 Receptor is Expressed in Vascular Endothelium and Maintained After Ischemia

To determine the cellular localization of LPA receptor expression in the brain and identify the receptor subtype likely mediating the protective effects of LPA, we performed a comprehensive expression analysis of LPA receptors across major brain cell populations and vascular compartments. Single-cell analysis revealed distinct expression patterns of different LPA receptor subtypes (Fig. 5E). UMAP visualization of cell clusters showed major brain cell types, including Oligo, Astro, neurons, MG, ECs, ependymal cells, and additional supporting populations (Fig. 5E, left panel).

Analysis of receptor expression patterns revealed that the LPA4 receptor was prominently and selectively expressed in vascular EC (Fig. 5F). Feature plots confirmed enrichment of LPA4 expression within the endothelial cell cluster, consistent with its putative role in regulating BBB integrity. The identity of each cluster was verified using established cell-type marker genes, including *Cldn5* for EC, *Tmem119* for microglia, and *Mbp* for oligodendrocytes (Fig. 5F). Comparison between sham and ischemic conditions demonstrated that endothelial LPA4 expression was preserved following ischemic injury (Fig. 5G). The maintenance of LPA4 expression in post-ischemic vessels suggests that EC retain LPA4 receptor availability after stroke, consistent with the potential role of LPA4 in mediating BBB protection after ischemia.

## Discussion

In this study, we show that LPA protects against BBB disruption and mitigates ischemic brain injury following focal cerebral ischemia in mice. LPA administration significantly reduced infarct volume, attenuated brain edema, preserved BBB integrity, and showed trends toward improved behavioral outcome after dMCAO. Notably, LPA did not reduce BBB permeability and infarct volume in LPA4 KO mice; this finding highlights LPA4 as a crucial mediator of the above-mentioned protective effects on vascular permeability and infarct size. Transcriptomic analysis further revealed that LPA induces distinct shifts in gene expression profiles favoring vascular stabilization and anti-inflammatory responses. Additionally, single-cell analysis confirmed LPA4 receptor expression in vascular endothelial cells, which was sustained after ischemia. These findings suggest LPA–LPA4 signaling axis as a potential therapeutic target for preserving cerebrovascular function after ischemic stroke.

BBB disruption is a major determinant of secondary injury after ischemic stroke, driving vasogenic edema, inflammatory cell infiltration, and worsening neurological outcomes [3]. Consistent with this, our findings showed that LPA treatment significantly reduced Evans Blue and FITC-dextran extravasation, demonstrating effective preservation of BBB integrity. LPA reduced vascular permeability in both the ischemic core and penumbra, indicating spatially broad stabilization of the neurovascular unit. This protective effect correlated with preserved claudin-5 expression in endothelial cells, suggesting that LPA helps maintain tight junction architecture that is otherwise compromised during ischemia. Additional studies are required to directly assess whether LPA4 specifically mediates the preservation of claudin-5 expression in LPA4 KO animals; however, LPA4 couples primarily to Gαs and Gαi proteins, modulating cyclic AMP (cAMP) signaling [27], which is known to enhance endothelial barrier function through protein kinase A (PKA)-dependent stabilization of tight junctions. LPA signaling can also activate Rho family GTPases, key regulators of cytoskeletal organization and tight junction assembly [48, 49]. These mechanisms provide a plausible mechanistic basis for LPA-mediated tight junction preservation, although direct evidence linking LPA4 signaling to maintenance of claudin-5 expression requires further investigation in LPA4-deficient animals.

The essential role of LPA4 in these protective effects was confirmed in LPA4 KO mice, where LPA failed to reduce BBB leakage or infarct size. Furthermore, single-cell expression analyses revealed prominent LPA4 receptor expression in vascular endothelial cells, the principal structural component of the BBB, and demonstrated that this expression is maintained after ischemic injury. Although our findings strongly support an endothelial mechanism, we cannot exclude contributions from other cell types expressing lower levels of LPA4, especially given the diverse and context-dependent effects of LPA receptors in CNS disorders [11]. For example, LPA1 has been implicated in promoting BBB disruption in neuroinflammatory conditions [50], whereas our results highlight an opposing, receptor-specific protective role for LPA4.

Several previous studies have reported deleterious effects of LPA on ischemic brain injury and BBB disruption [24–26, 51, 52], predominantly implicating the autotaxin–LPA axis or LPA1/LPA5 signaling. The apparent discrepancy with our findings likely reflects the context dependence of LPA biology, including differences in receptor subtype engagement, dose, and model systems. Notably, the protective effects in the present study were strictly LPA4-dependent, as confirmed from their abolition in LPA4 KO mice. The observed preservation of MAP2-positive neurons in regions with reduced FITC-dextran leakage in LPA-treated mice suggests that the effects of LPA extend beyond the endothelium to support the broader neurovascular unit. This finding aligns with the evidence that BBB integrity is essential for neuronal survival following ischemia, and supports the concept that interventions that preserve BBB function can confer indirect neuroprotection [4].

Our findings have several implications for therapeutic development. Current neuroprotective strategies for ischemic stroke have had limited clinical success [53], in part because many approaches fail to address BBB dysfunction—a major contributor to poor outcomes and hemorrhagic transformation following reperfusion therapy [54, 55]. The observation that LPA provides neuroprotection when administered immediately after ischemia onset suggests that LPA4 stimulation could complement existing therapies, including thrombolysis and thrombectomy, by reducing reperfusion-induced BBB breakdown. Given that different LPA receptors can mediate opposing effects in the CNS, receptor selectivity is crucial for therapeutic development. The requirement for LPA4 in mediating LPA-induced reduction in vascular permeability and infarct volume, as demonstrated by the results of LPA4 KO experiments, highlights the potential utility of developing selective LPA4 agonists, which may offer improved safety compared with nonselective LPA receptor agonists [21–23]. Third, because ischemia paradoxically exacerbates BBB breakdown during reperfusion [56], LPA could be combined with existing reperfusion therapies to provide additive protection by stabilizing the BBB and mitigating reperfusion injury, potentially improving outcomes in patients undergoing thrombolysis or thrombectomy.

Despite these promising results, our study has some limitations. First, LPA was administered immediately following dMCAO, and the therapeutic time window for LPA activation remains undefined. Future studies to evaluate the effects of delayed LPA administration are essential to determine the clinical applicability of the findings.

Second, the present study employed a permanent dMCAO model, which does not recapitulate reperfusion injury occurring in patients treated with thrombolysis or thrombectomy. Permanent dMCAO was selected because of its surgical reproducibility and low inter-animal variability; however, reperfusion introduces additional pathological mechanisms, including reactive oxygen species generation and matrix metalloproteinase activation, that might differentially engage LPA receptor signaling. Future studies using transient MCAO or embolic models are warranted to assess LPA–LPA4 signaling in the setting of reperfusion injury.

Third, we focused primarily on endothelial mechanisms, and contributions from astrocytes, pericytes, or infiltrating immune cells—each of which influences BBB function—were not examined in detail.

Fourth, behavioral assessment was limited. Although the cylinder test is sensitive for detecting forelimb asymmetry after cortical stroke, it does not capture the full spectrum of neurological deficits. Future studies incorporating additional behavioral paradigms, such as the rotarod, adhesive removal test, and grid walk, would enable a more comprehensive assessment of neuroprotection.

Fifth, only adult female mice were used, and estrous cycle stage was not controlled. As hormonal status can influence BBB integrity and stroke severity, the generalizability of the findings to male animals and different hormonal states remains uncertain. Potential sex differences in the LPA–LPA4 axis warrant investigation in future studies.

Sixth, all outcomes were assessed at 48 h post-ischemia, and the longer-term effects of LPA treatment on infarct maturation, neurological recovery, and potential risks of delayed secondary injury were not examined.

Seventh, the dose of LPA used (10 mg/kg) was selected based on preliminary comparisons of 3, 5, and 10 mg/kg doses without performing a full dose-response analysis. As LPA acts as a non-selective agonist across multiple LPA receptor subtypes, the receptor selectivity of LPA at this dose in vivo cannot be guaranteed. Although the loss of protection in LPA4 KO mice indicates that LPA4 signaling is required for the observed effects, we cannot exclude contributions from other receptors in wild-type animals.

Eighth, BBB permeability measurements were not normalized to infarct volume, which limited our ability to conclusively distinguish a direct BBB-stabilizing effect from secondary reduction in permeability resulting from smaller infarcts.

Finally, pharmacokinetic and safety profiling of LPA or LPA4-selective agonists were not evaluated in this study; such analyses will be required to assess the translational feasibility of targeting LPA–LPA4 signaling.

In conclusion, this study identifies LPA–LPA4 signaling as a previously unrecognized mechanism for preserving BBB integrity and mitigating cerebral ischemia. LPA treatment reduces infarct volume and brain edema accompanied by preserved claudin-5 expression and modulation of vascular stabilization and anti-inflammatory transcriptional responses. LPA4 KO experiments establish that LPA4 is required to reduce vascular permeability and infarct volume, and single-cell analysis demonstrates selective LPA4 expression in vascular endothelial cells together with its robust endothelial expression and sustained presence after ischemia, provides a compelling rationale for consideration of LPA4-targeted therapeutic strategies. Given the persistently high global burden of stroke-related disability and mortality, and the paucity of effective neuroprotective therapies, the LPA–LPA4 pathway represents a promising target for further translational investigation.

## Data Availability

The data supporting the findings of this study are available from the corresponding author upon reasonable request. RNA-seq datasets generated for this work will be deposited in the Gene Expression Omnibus (GEO) database upon acceptance of the manuscript. In addition, the publicly accessible dataset GSE261494 was utilized to assess the expression of LPA receptors in the mouse cortex.
